# Aggressive (deep) angiomyxoma of the vulva: a case report 

**DOI:** 10.1186/s13256-022-03284-z

**Published:** 2022-02-18

**Authors:** Lajya Devi Goyal, Priyanka Garg, Rama Badyal, Shivali Bhalla

**Affiliations:** 1 Department of Obstetrics and Gynecology, All India Institute of Medical Sciences, Bathinda, Punjab 151001 India; 2 Department of Pathology, Government Medical College, Amritsar, Punjab 143001 India; 3 Department of Obstetrics and Gynecology, Guru Gobind Singh Medical College, Faridkot, Punjab 151203 India

**Keywords:** Angiomyxoma, Vulva, Gynecology, Local recurrence, GnRH agonist

## Abstract

**Background:**

Aggressive angiomyxoma of the vulva is a benign, slow-growing tumor originating from myxoid cells of connective tissue. The tumor is known for multiple local recurrences with a low tendency to metastasize. Only around 350 cases have been documented in the scientific literature so far.

**Case presentation:**

We report a case of a 40-year-old North Indian, unmarried woman with a swelling on the left labium majora for 7 years, along with surface ulceration over the mass. Local examination showed a well-circumscribed, 8 × 8 cm pedunculated  mass arising from the left labium majora with an overlying ulcer measuring 6 cm × 4 cm. After taking informed written consent, wide local excision of the mass and surrounding margins was done under anesthesia. Histopathology was suggestive of aggressive angiomyxoma. Immunohistochemistry was done, which was positive for estrogen and progesterone receptors. Her postoperative recovery was uneventful. The patient was given three doses of gonadotropin-releasing hormone agonist (injection, leuprolide 3.75 mg) once a month. No recurrence has been reported so far on follow-up visits for 1 year.

**Conclusion:**

Aggressive angiomyxoma is one of the differential diagnoses for vulvovaginal growth in a female. As the tumor is well known for local recurrences, correct diagnosis and appropriate management using a multidisciplinary approach are crucial to managing such patients.

## Introduction

Aggressive angiomyxoma (AA) of the vulva is a benign, slow-growing, and locally invasive mesenchymal tumor originating from myxoid cells of connective tissue. It was first described in a case series of nine patients by Steeper and Rosai in 1983 [1]. The term was reclassified as deep angiomyxoma by the World Health Organization in 2003 [2]. It is a relatively rare tumor, with less than 350 cases reported to date [3]. AA occurs almost exclusively in women of reproductive age, with a peak incidence in the third to fifth decades of life [4]. It usually presents as a painless mass in the vulvoperineal region. The word “aggressive” denotes its high tendency for local invasion and propensity to infiltrate the perivaginal and pararectal tissues. While mostly benign, the tumor is notorious for multiple local recurrences as high as 30–72% [5]. However, the tendency to metastasize is low. Due to the rarity of this tumor, the chances of misdiagnosis vary between 70% and 100%, leading to improper and delayed treatment that adversely affects the patient’s health. Wide local excision with negative margins and long-term follow-up remains the best course of management but often results in high morbidity. Adjuvant therapy in the form of gonadotropin-releasing hormone (GnRH) agonists has shown promising results to prevent recurrences. We discuss a case of AA of the vulva that was successfully managed by surgical excision followed by injection of leuprolide to avert relapse of the tumor, and present a brief literature review.

## Case presentation

**Fig. 1:**
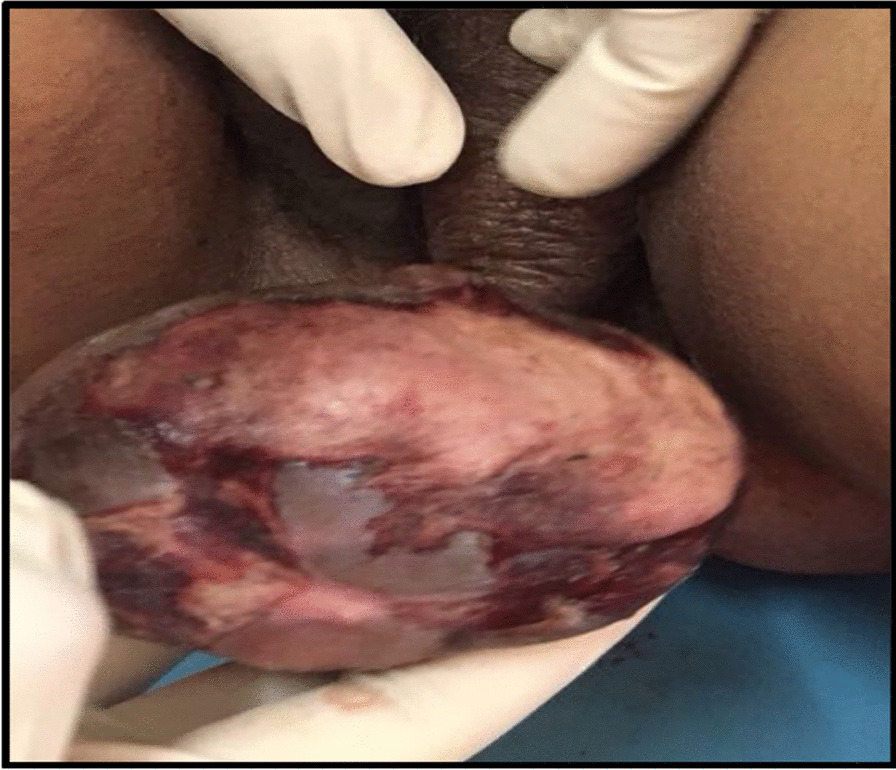
A large, well-defined pedunculated mass arising from left labium majora with overlying skin ulceration

**Fig. 2:**
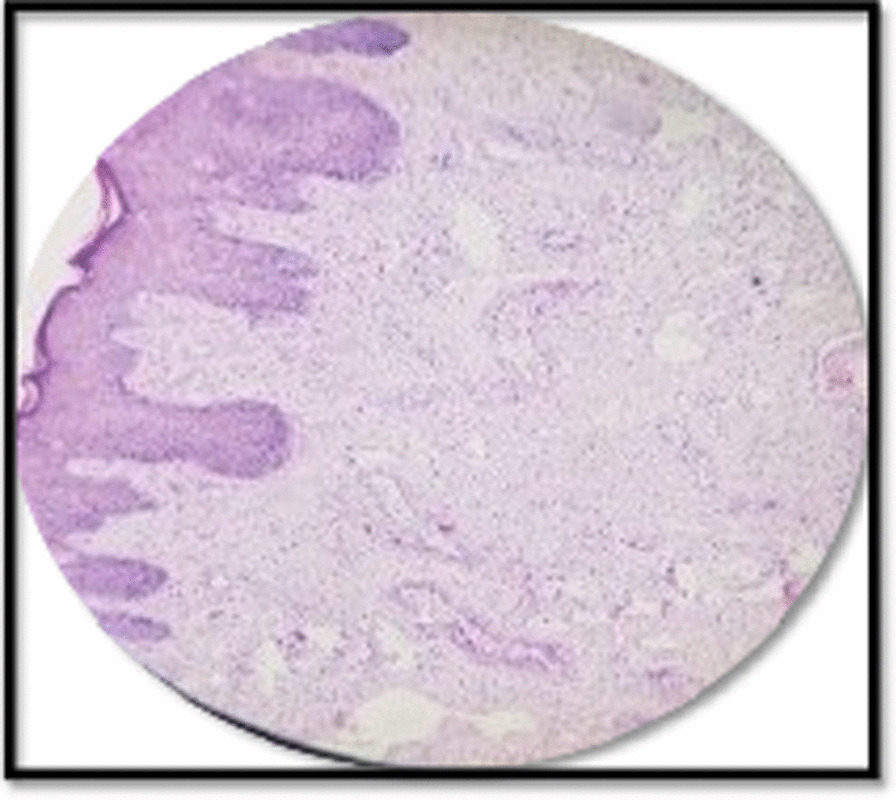
Histological section [hematoxylin and eosin (H&E), ×100] showing blood vessels of varying size against myxoid background

A 40-year-old North Indian, unmarried, nulliparous woman came to gynecology Out Patient Department (OPD) with a swelling on the left labium majora for 7 years, slowly growing in size. There was ulceration over the mass associated with serosanguinous discharge. Past medical and family history was unremarkable. On general examination, the patient was moderately built and afebrile. There was no evidence of jaundice, anemia, cyanosis, lymphadenopathy, clubbing, weight loss, or any bowel and bladder function alterations. Her menstrual cycles were regular with normal flow. She was a known case of epilepsy and was on tablet levetiracetam 500 mg once daily. She had a history of hemithyroidectomy 6 years back and was on thyroxine tablet 50 μg once daily. Her abdomen was soft and nontender. On local examination, a well-circumscribed, 8 × 8 cm pedunculated mass arose from left labium majora lateral to introitus at five o’clock position. On palpation, the mass was nontender, nonreducible, and soft in consistency. There was an overlying ulcer measuring 6 cm × 4 cm, and the floor was covered with unhealthy pale granulation tissue. There was mild serosanguinous discharge from the ulcer (Fig. 1). No inguinal lymphadenopathy was seen. On gynecological examination, the uterus was normal in size with a healthy cervix and vagina. Her baseline investigations were normal. Ultrasonography (USG) of the abdomen and pelvis was normal. Magnetic resonance imaging (MRI) could not be done due to financial constraints on the part of the patient. After taking informed written consent, wide local excision of the mass and surrounding margins was done under anesthesia. There was a moderate amount of bleeding during the surgery. The specimen was sent for histopathological examination. On histopathology, the tumor was composed of spindle and stellate-shaped cells in a myxoid matrix suggestive of aggressive angiomyxoma with tumor free margins (Fig. 2). Immunohistochemistry was done, which was positive for estrogen and progesterone receptors. Her postoperative recovery was uneventful. On the third day post-surgery, the patient was sent home in good general condition, with hemoglobin of 10 gm%. Due to the high propensity of the tumor for local recurrences, the patient was given three doses of GnRH agonist (injection, leuprolide 3.75 mg) once a month. No recurrence has been reported so far on follow-up visits for 1 year.

## Discussion

Angiomyxomas are recognized as superficial (cutaneous myxoma) or aggressive. Superficial angiomyxomas occur more commonly in middle-aged patients presenting as a single nodule or a polypoidal mass in the head and neck region, trunk, and lower extremities. They are easily confused with skin tags, cysts, or neurofibroma and are commonly associated with Carney’s complex (a triad of cardiac myxomas, spotty pigmentation, and endocrine overactivity). AA is reported almost exclusively in women of reproductive age, with occasional cases seen in perimenopausal females and children. This may be attributed to the hormone-responsive nature of the tumor, as its growth is stimulated by estrogen and progesterone, backed by a case report of rapid enlargement during pregnancy [6]. The tumor is located mainly in the pelvis and perineal region. Other rare sites include lung, liver, larynx, and orbit [3]. Occasionally, they are seen in male patients in related sites like the scrotum and inguinal region. However, the ratio of occurrence in females to males is 6:1 [7].

The exact pathogenesis of AA is still unclear. However, since the tumor cells express desmin and smooth muscle actin, it is believed to originate either from specialized mesenchymal cells or from the multipotent perivascular progenitor cells [8]. High-mobility group protein isoform I-C (*HMGI-C*) gene located in the region 12q13-15 of chromosome 12 seems to play a role in the pathogenesis of this tumor. Abnormal expression of this gene, *HMGI-C*, detected using the immunoperoxidase technique, may be a possible marker of microscopic residual disease [9].

The patient is usually asymptomatic and presents with a slow-growing pedunculated mass in the vulva, gluteal region, or suprapubic region. Nonspecific symptoms such as dull aching pain, the feeling of local pressure, dysuria, urinary retention, or dyspareunia may be present. The size may range from a few centimeters to > 60 cm. The true extent of AA is frequently underrated on gross inspection as the clinically visible tumor exhibits only a fragment of the more widespread involvement of the deeper tissues of the pelvis and retroperitoneum.

Clinically, the tumor needs to be differentiated from angiomyofibroblastoma, Bartholin gland cyst, sarcoma botryoides, superficial angiomyxoma, vulvar hypertrophy with lymphedema, lipoma, and sarcoma. The histological differential diagnosis includes myxoma, myxoid liposarcoma, myxofibrosarcoma, and nerve sheath myxoma. Marked vascularity in aggressive angiomyxoma helps to differentiate it from most of the above neoplasms. The absence of lipoblasts differentiates it from liposarcoma. AA may be commonly misdiagnosed as angiomyofibroblastoma. However, the latter is well circumscribed, more cellular, and more vascular.

Preoperative imaging is extremely important to see the extent of the tumor and plan surgical excision accordingly. On USG, it is seen as a hypoechoic or cystic mass. The mass shows a distinct feature of swirled and layered tissue on computed tomography (CT) and magnetic resonance imaging (MRI). Wu *et al.* conducted a study to evaluate the role of CT and MRI imaging techniques. They elucidated that both CT and MRI precisely predict the extent of the tumor. However, MRI is more specific and is superior to CT when ascertaining the tumor’s relation to the surrounding structures [9]. Thus, MRI is the investigation of choice for diagnosis and follow-up of recurrences.

Many treatment modalities have been tried with varying success. Radical surgical excision with negative margins is the conventional treatment of choice. However, it is not always possible to achieve negative resection margins as the tumor is locally infiltrative, leading to high operative morbidity. Therefore, less radical surgery is recommended nowadays. Adjuvant therapy in raloxifene, tamoxifen, or GnRH agonists like leuprolide acetate and goserelin have proven beneficial where the tumor is estrogen and progesterone receptor sensitive. In a case report by Fine *et al.*, recurrent AA of the vulva was treated solely by 3 months of GnRH agonist without needing any other medical therapy or surgery [10]. However, its long-term use may not be acceptable to the patients because of unwanted side effects (menopausal symptoms and bone loss). The diagnosis of AA in our patient was elucidated after the histopathology report, so we administered three doses of leuprolide injection for any remnant disease and avoided recurrence. Radiotherapy and chemotherapy have a limited role owing to the low mitotic activity of the tumor. AA is known for multiple local recurrences to the extent of 36–72% and may occur as early as 2 months to as late as 20 years [11]. Begin *et al.* described nine AA cases with local recurrence, all due to incomplete excision [12]. Han-Guerts *et al.* suggested multimodality treatment for AA: excision of the tumor without mutilation, adjuvant therapy such as arterial embolization or hormonal therapy, and radiotherapy in symptomatic patients resistant to embolization/hormonal therapy.

The tumor was conventionally considered to be nonmetastasizing. However, metastasis has been reported as an exceedingly rare event in literature. Siassi *et al.* reported a death due to multiorgan metastasis invading the peritoneum, lungs, and lymph nodes [13]. In another case, it occurred in a 34-year-old woman who developed several local recurrences after primary resection of an AA and subsequently died from multiple lung metastases [11]. No evidence-based recommendations are available for post-surgery management of AA, but due to the high rate of local recurrences and possible metastasis, patients should be advised to undergo long-term follow-up until 15 years after the primary excision.

## Conclusion

Aggressive angiomyxoma is a rare, locally aggressive neoplasm. It should be borne in mind as a differential diagnosis whenever a patient presents with growth in the vulvovaginal region, perineum, or pelvis. As the tumor is well known for local recurrences, timely diagnosis and management with surgical excision and adjuvant therapy are beneficial for such patients.

## Data Availability

Not applicable.
